# Effect of Green Tea Mouthwash on Oral Malodor

**DOI:** 10.5402/2013/975148

**Published:** 2012-12-02

**Authors:** Supanee Rassameemasmaung, Pakkarada Phusudsawang, Vanida Sangalungkarn

**Affiliations:** ^1^Department of Oral Medicine and Periodontology, Faculty of Dentistry, Mahidol University, Yothi Road, Ratchathewi, Bangkok 10400, Thailand; ^2^Dental Division, Kanchanaburi Municipality, Lak Muang Road, Amphur Muang, Kanchanaburi 71000, Thailand; ^3^Department of Pharmacology, Faculty of Dentistry, Mahidol University, Yothi Road, Ratchathewi, Bangkok 10400, Thailand

## Abstract

This study aimed to determine the effect of green tea mouthwash on oral malodor, plaque, and gingival inflammation. Gingivitis subjects who had over 80 parts per billion of volatile sulfur compounds (VSC) in the morning breath were randomly assigned into green tea or placebo mouthwash group. At baseline, VSC, Plaque Index (PI) and Papillary Bleeding Index (PBI) were recorded. Participants were rinsed with the assigned mouthwash, and VSC level was remeasured at 30 minutes and 3 hours postrinsing. For the following 4 weeks, participants were asked to rinse with the assigned mouthwash twice daily. VSC, PI and PBI were remeasured at day 28. It was found that, at 30 minutes and 3 hours postrinsing, VSC was reduced by 36.76% and 33.18% in the green tea group and 19.83% and 9.17% in the placebo group, respectively. At day 28, VSC was reduced by 38.61% in the green tea group and 10.86% in the placebo group. VSC level in the green tea group was significantly different when compared to the placebo. PI and PBI were significantly reduced in both groups. However, no significant difference was found between groups. In conclusion, green tea mouthwash could significantly reduce VSC level in gingivitis subjects after rinsing for 4 weeks.

## 1. Introduction

Oral malodor is defined as an unpleasant breath odor whose causes originate from the mouth. This condition is caused mainly by the emanation of volatile sulfur compounds (VSC) especially hydrogen sulfide, methyl mercaptan, and dimethyl sulfide through the mouth air. These foul-smelling compounds are produced through the degradation of sulfur-containing amino acids by anaerobic Gram-negative bacteria. Other odoriferous compounds, for example, indole and cadaverine are also reported to cause oral malodor [1].

Oral malodor was found to correlate with periodontal disease. Quirynen et al. [2] found that 11% of 2,000 patients who suffered from bad breath had periodontal health problem. Takeuchi et al. [3] reported a positive association between periodontal parameters and the severity of oral malodor. The increase in oral malodor in subjects with periodontal disease is attributed to a higher number of Gram-negative, periodontal bacteria within the oral cavity. These bacteria including *Porphyromonas gingivalis*, *Prevotella intermedia*, *Fusobacterium nucleatum*, *Tannerella forsythia*, and *Eubacterium* species and spirochetes are known to produce significant amount of VSC [4]. In addition, desquamated cells or blood elements from the inflamed gingival tissue can provide sources for microbial putrefaction. In contrast, oral malodor was found to decrease after subsidence of gingival inflammation [3].

Mechanical cleaning methods including tooth brushing and tongue scraping have been found to reduce oral malodor [5]. However, such methods are not adequately practiced by large number of individuals. Therefore, the use of mouthwash is provided as an alternative approach [6, 7]. The mechanism of mouthwash to reduce odor intensity occurs through an antimicrobial activity against oral bacteria or the conversion of VSC into nonodorous compounds [1].

*Camellia sinensis*, or Green tea, has a wide variety of pharmacological activities [8–13]. Green tea contains polyphenols especially the major four catechins, that is, (-)-epigallocatechin gallate (EGCG), (-)-epicatechin gallate (ECG), (-)-epicatechin (EC), and (-)-epigallocatechin (EGC) [14]. Catechins showed an *in vitro* bactericidal activity against odor-producing, periodontal bacteria, *P. gingivalis*, and *Prevotella* spp., with an MIC of 1.0 mg/mL [8]. Green tea also inhibited adherence of *P. gingivalis* to oral epithelial cells at a concentration below 0.25 mg/mL [9]. Catechins and its derivatives could reduce periodontal breakdown by inhibiting collagenase [10] and cysteine proteinase activity of *P. gingivalis *[11]. EGCG has an inhibitory effect on protein tyrosine phosphatases (PTPase) activity in *P. intermedia* [12]. Immediately after administered, green tea powder could reduce VSC concentration in the mouth air [13]. Green tea could also inhibit VSC production in a saliva-putrefaction system [13]. In this study, green tea mouthwash was formulated. We hypothesized that green tea mouthwash could reduce oral malodor after one-time usage and could reduce oral malodor as well as gingival inflammation after 4-week usage in gingivitis subjects. Thus, the objectives of this study were to determine the effect of green tea mouthwash on the level of VSC after one-time usage and on the level of VSC, plaque and gingival inflammation after 4-week usage in gingivitis subjects.

## 2. Method

This study was a double-blinded and placebo-controlled clinical trial. The study design was followed to the American Dental Association guideline for the assessment of efficacy of oral malodor products [15]. The study protocol was documentary proved by Mahidol University Institutional Review Board (COA. no. MU-IRB 2008/177.1211) and registered in ClinicalTrials.gov (NCT00932347). The study was performed at the Postgraduate Clinic, Faculty of Dentistry, Mahidol University, Bangkok, Thailand.

Green tea mouthwash and placebo mouthwash were prepared from the Department of Pharmacology, Faculty of Dentistry, Mahidol University. Green tea mouthwash was a hydroalcoholic brownie solution. It contained green tea extract, propylene glycol, parabens, saccharin, and mint flavor. The green tea extract had more than 80% of total catechins. The placebo mouthwash was a hydroalcoholic brownie solution. It contained the same ingredients but without green tea extracts.

### 2.1. Participants

Sixty participants were recruited from gingivitis patients at the Faculty of Dentistry, Mahidol University. Those who had at least 20 teeth and had over 80 parts per billion (ppb) of VSC in the mouth air were invited to enroll in the study. Patients were excluded from the study if they had systemic complicating factors or oral mucosal lesions. They were also excluded from the study if they were smokers, were denture wearers, or took an antibiotic 1 month prior to the study. Before entering the study, informed consents were obtained from all participants.

### 2.2. Sample Size Calculation

Number of participants was calculated according to the level of VSC (primary outcome measure) after using 0.2% chlorhexidine mouthwash or 0.03% triclosan mouthwash [16]. We calculated the sample size as follows:

(1) N(per  group)=2(Zα/2+Zβ)2σ2(X−1−X−2)2.

A sample size of at least 25 participants in each group would provide 80% power of detection at a two-sided 5% significance level. Thirty participants were enrolled in each group to compensate for the drop out participants.

### 2.3. Screening Examination

Screening examination was done between 7.00–8.30 a.m. Patients were asked to refrain from tooth brushing, mouth rinsing, eating, or drinking for at least 2 hours prior to the measurement. They were asked to rinse with water for at least 20 minutes before the measurement to protect dry mouth effect and stop talking for at least 5 minutes. VSC level was measured by portable sulfide monitor (RH-17, Halimeter, Interscan Corp, CA, USA) as described by the manufacturer. The measurements were performed 3 times, and the mean levels of VSC were recorded as peak ppb. Only gingivitis patients who had over 80 ppb of VSC were invited to enroll the study.

### 2.4. Experimental Procedure

After screening examination, participants were stratified according to their VSC level. The participants in each strata were randomly allocated into the green tea mouthwash group (mouthwash containing *C. sinensis* extracts) or the placebo mouthwash group using sealed envelopes. Baseline parameters consisting of VSC level, Plaque Index; PI [17], and Papillary Bleeding Index; PBI [18] were recorded by one examiner (P. Phusudsawang) who was blind to the allocation until data collection was completely done. Participants were asked to refrain from tooth brushing, mouth rinsing, eating, or drinking for at least 2 hours prior to the measurement. They were asked to rinse with water for at least 20 minutes before the measurement to protect dry mouth effect and stop talking for at least 5 minutes. VSC level was measured between 7.00–8.30 a.m. by the sulfide monitor as previously described. Subsequently, PI and PBI were scored on the Ramfjord teeth. Results were recorded as means of the buccal and lingual surfaces examined. The participants were thoroughly rinsed with 15 mL of the assigned mouthwash for 1 minute. To determine the effect of mouthwash on oral malodor after one time usage, the level of VSC was remeasured at the following 30 minutes and 3 hours. Within this period, participants were refrained from tooth brushing, rinsing, eating, or drinking.

**Figure 1:**
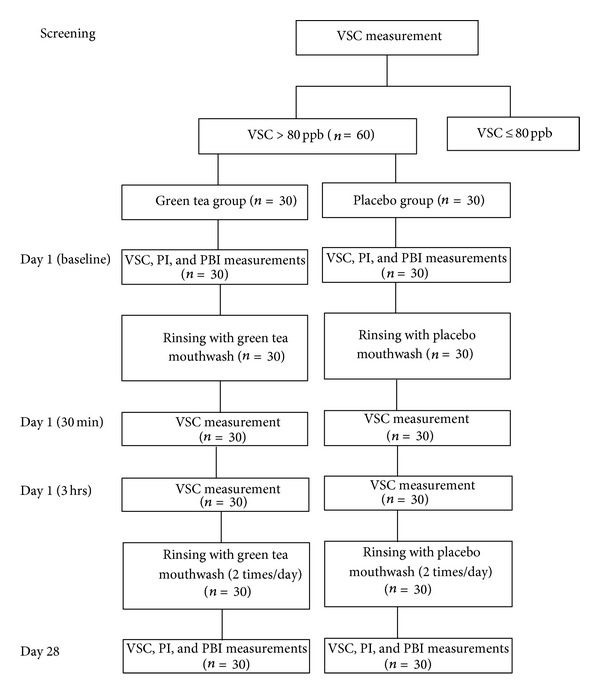
Experimental procedure.

To determine the effect of mouthwash on oral malodor and plaque and gingival inflammation after 4-week usage, the participants were asked to rinse with the assigned mouthwash twice daily after tooth brushing and were asked to refrain from drinking or water rinsing for at least 30 minutes after mouthwash usage. At day 28, VSC, PI, and PBI were recorded again as previously described (Figure 1).

During the course of the study, participants were asked to use the assigned toothbrush and toothpaste (M-Dent, Mahidol University) and continued their usual oral hygiene and dietary habits but refrain from rinsing with other mouthwash.

### 2.5. Evaluation of Participants' Compliance and Side Effects

At day 14 and day 28, participants were asked to return the bottles of the assigned mouthwash. The remaining content of mouthwash was measured. At day 28, they were also asked to complete the questionnaire regarding xerostomia, burning sensation, and changes in taste perception and received oral examination to determine tooth staining and other signs of mucosal irritation. At the end of the experiment, oral hygiene instruction and oral prophylaxis were given to all participants.

### 2.6. Statistical Analysis

For the comparison within group, paired *t*-test was used to analyze the VSC level between baseline and 30 minutes, baseline and 3 hours, as well as baseline and day 28, while Wilcoxon sign rank test was used to analyze PI and PBI between baseline and Day 28. For the comparison between green tea mouthwash group and placebo mouthwash group, *t*-test was used to compare VSC level, and Mann-Whitney *U* test was used to compare PI and PBI. The significance level was set at *P* < 0.05.

## 3. Results

### 3.1. Study Population

Sixty participants completed the study. There was no dropout participant. The green tea mouthwash group comprised of 30 participants (27 women and 3 men) with an age range between 18–55 years (mean age of 27.2 ± 9.1 years). The placebo mouthwash group comprised of 30 participants (27 women and 3 men) with an age range between 19–42 years (mean age of 25.8 ± 7.6 years). During the experimental period, none of them received medical interventions which might influence the outcome of the study. At baseline, VSC, PI, and PBI were not significantly different between groups.

### 3.2. Effect of Green Tea Mouthwash on Level of Volatile Sulfur Compounds (VSC)

**Figure 2:**
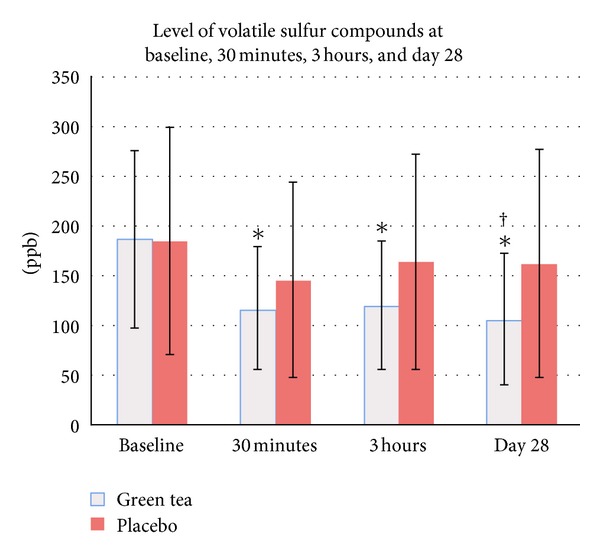
Level of volatile sulfur compounds before and after mouthwash usage: ∗ significantly different compared to baseline (*P* < 0.05) and † significantly different compared to placebo (*P* < 0.05).

At baseline, VSC level in the green tea group and the placebo group was 187.7 ± 90.3 ppb (95% CI: 155.4–220 ppb) and 185.3 ± 115.1 ppb (95% CI: 144.1–226.4 ppb), respectively. After one-time usage, VSC level was reduced to 116.6 ± 63.6 ppb (95% CI: 93.8–139.4 ppb) at 30 minutes and 119.6 ± 65.4 ppb (95% CI: 96.2–143 ppb) at 3 hours in the green tea group and 146.2 ± 99.6 ppb (95% CI: 110.6–181.9 ppb) at 30 minutes and 164.6 ± 109.3 ppb (95% CI: 125.5–203.7 ppb) at 3 hours in the placebo group (Figure 2). The reduction of VSC at 30 minutes and 3 hours postrinsing was 36.76 ± 22.00% and 33.18 ±32.29% in the green tea group and 19.83 ± 25.25% and 9.17 ±  27.81% in the placebo group, respectively. Compared to baseline, VSC level was significantly different in the green tea group at 30 minutes (*P* < 0.0005) and 3 hours (*P* < 0.0005) postrinsing. It appeared that the green tea mouthwash could more effectively reduce VSC level when compared to the placebo mouthwash; however, no significant difference was found between groups at any time points (*P* = 0.175 at 30 minutes; *P* = 0.058 at 3 hours).

At day 28, VSC level was 105.5 ± 66.6 ppb (95% CI: 81.6–129.3 ppb; 38.61 ± 36.18% reduction from baseline) in the green tea group and 162.4 ± 115.7 ppb (95% CI: 121–203.8 ppb; 10.86 ± 25.40% reduction from baseline) in the placebo group (Figure 2). VSC level in the green tea group was significantly different when compared to baseline (*P* < 0.0005) and to the placebo group (*P* = 0.023).

### 3.3. Effect of Green Tea Mouthwash on Plaque Index (PI) and Papillary Bleeding Index (PBI)

**Figure 3:**
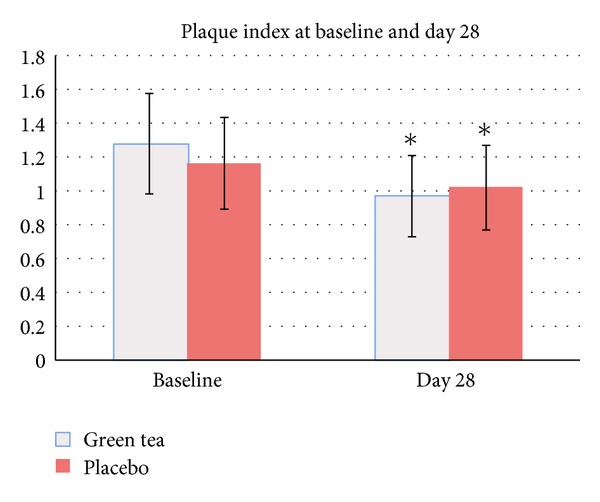
Plaque Index before and after mouthwash usage: ∗ significantly different compared to baseline (*P* < 0.05).

PI in the green tea mouthwash group and the placebo mouthwash group at baseline was 1.29 ± 0.30 (95% CI: 1.18–1.40) and 1.17 ± 0.27 (95% CI: 1.07–1.27), respectively. After day 28, PI was reduced to 0.97 ± 0.24 (95% CI: 0.88–1.06; 24.8% reduction from baseline) in the green tea mouthwash group and 1.02 ± 0.25 (95% CI: 0.93–1.11; 12.82% reduction from baseline) in the placebo mouthwash group (Figure 3). PI was significantly different when compared to baseline in the green tea mouthwash group (*P* = 0.001) and the placebo mouthwash group (*P* = 0.001). However, there was no significant difference between groups (*P* = 0.37).

**Figure 4:**
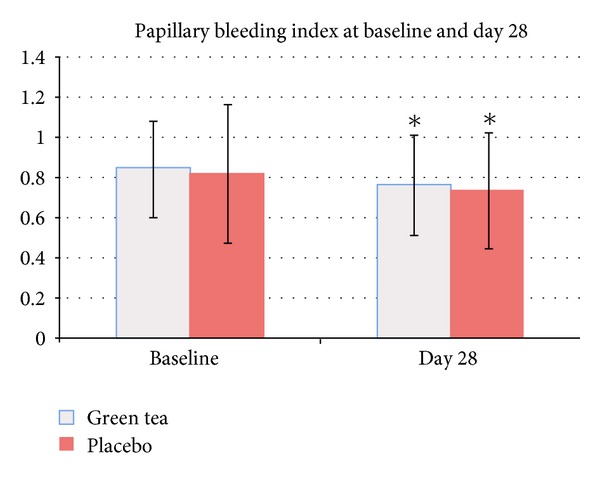
Papillary Bleeding Index before and after mouthwash usage: ∗ significantly different compared to baseline (*P* < 0.05).

PBI in the green tea mouthwash group and the placebo mouthwash group at baseline was 0.85 ± 0.24 (95% CI: 0.76–0.94) and 0.82 ± 0.35 (95% CI: 0.69–0.95), respectively. At day 28, PBI was 0.76 ± 0.25 (95% CI: 0.67–0.85; 10.58% reduction from baseline) in the green tea mouthwash group and 0.73 ± 0.29 (95% CI: 0.63–0.83; 10.97% reduction from baseline) in the placebo mouthwash group (Figure 4). PBI at day 28 was significantly different when compared to baseline in the green tea mouthwash group (*P* = 0.001) and the placebo mouthwash group (*P* = 0.045). However, there was no significant difference between groups (*P* = 0.677).

### 3.4. Evaluation of Participants' Compliance and Side Effects

The remaining content of the mouthwash was not different at day 14 and day 28 in both groups (data not shown) and thus indirectly indicated good participants' compliance. In this study, tooth staining, oral tissue irritation and desquamation, and burning sensation as well as abnormality in the taste perception after using green tea mouthwash or placebo mouthwash for 4 weeks were not found.

## 4. Discussion

The pharmacological activity of green tea has been well documented in the field of dentistry [8–12]. In this study, green tea mouthwash was formulated, and its effect on oral malodor was assessed at different periods after applying. At 30 minutes and 3 hours postrinsing, green tea mouthwash could reduce VSC level by 36.76% and 33.18%, respectively. The level of VSC at 30 minutes and 3 hours postrinsing was significantly different when compared to baseline. However, no significant difference in the VSC level was found when compared to the placebo mouthwash. The reduction of VSC level after using green tea or placebo mouthwash may be explained by the action of rinsing which is capable of removing intraoral bacteria, desquamated cells, or tissue debris. Although green tea catechins can transform VSC to nonodorous compounds through the reaction with sulfhydryl and amino groups of VSC [19], this ability might be masked by the washout effect.

To assess the effect of green tea mouthwash on VSC level and gingival inflammation, the mouthwash was assigned to use twice daily for 4 weeks in gingivitis subjects. The result showed that VSC level was reduced by 38.61% after using green tea mouthwash for 4 weeks. Green tea mouthwash could significantly reduce VSC level when compared to baseline and to the placebo. The ability of green tea mouthwash in reducing oral malodor may result from an antimicrobial activity of green tea catechins, especially on *P. gingivalis* [8, 9] as well as the ability of green tea catechins to transform VSC to non-odorous compounds [19].

Regarding the effects of green tea mouthwash on plaque level, the reduction of plaque was found in both groups without significant difference between groups at the end of the experiment. This may be explained by the Hawthorne's effect. The reduction of VSC was not associated with the reduction of plaque in this study. Because plaque index used in this study could not detect qualitative difference in plaque, the effect of green tea mouthwash on certain periodontal bacteria needs to be further elucidated.

The interproximal area of the teeth is the area where plaque accumulation is commonly found. In this study, Papillary Bleeding Index (PBI) which reflected the condition of the interproximal marginal periodontium was used. At day 28, a significant reduction of PBI as compared to baseline was found in both groups. When compared between groups, significant difference in PBI could not be detected. It has been shown that catechins and its derivatives could inhibit tissue breakdown through the proteinase activity of *P. gingivalis* [10, 11]. In doing so, catechins and its derivatives could reduce source for microbial putrefaction. However, the effect of green tea mouthwash on the reduction of gingival inflammation could not be found in the present study. The time and the number of subjects enrolled in the study might be insufficient to detect the intergroup difference in PBI. Thus, the long-term clinical research involving large number of subjects may also be considered in the future studies.

Carvalho et al. [16] demonstrated that, in the absence of mechanical plaque control, rinsing with four commercially available mouthwash (0.05% cetylpyridinium chloride, essential oils, 0.03% triclosan, and 0.12% chlorhexidine gluconate) could significantly reduce VSC level by approximately 13%, 24%, 29%, and 62%, respectively. Winkel et al. [6] found that mouthwash containing 0.05% chlorhexidine, 0.05% cetylpyridinium chloride, and 0.14% zinc-lactate could reduce VSC level by 41%. According to the present study, green tea mouthwash demonstrated comparable percentage of VSC reduction to the mouthwash containing 0.05% chlorhexidine, 0.05% cetylpyridinium chloride, and 0.14% zinc-lactate but less percentage of VSC reduction compared to 0.12% chlorhexidine gluconate.

Oral malodor can be assessed by several methods. Organoleptic assessment (the direct sniffing of the exhaled air by human nose) most closely resembled daily situations in which malodor is detected. Due to its uncomfortability, the sulfide monitor was used in this study. Previous work by Rosenberg et al. [20] reported a significant correlation between the measurements obtained from the sulfide monitor and the organoleptic assessment. It should be noted that oral malodor is also caused, to a lesser extent, by other odoriferous compounds. The limitation of using the sulfide monitor is its inability to detect compounds other than VSC. Thus, the organoleptic assessment may be used in the future studies.

## 5. Conclusion

After using for 4 weeks, green tea mouthwash could significantly reduce VSC level in gingivitis subjects without causing remarkable side effects.
